# Impact of *Trypanosoma cruzi* on antimicrobial peptide gene expression and activity in the fat body and midgut of *Rhodnius prolixus*

**DOI:** 10.1186/s13071-016-1398-4

**Published:** 2016-03-01

**Authors:** CS Vieira, PJ Waniek, DP Castro, DP Mattos, OC Moreira, P Azambuja

**Affiliations:** Laboratório de Bioquímica e Fisiologia de Insetos, Instituto Oswaldo Cruz, Fundação Oswaldo Cruz (IOC/FIOCRUZ), Rio de Janeiro, RJ Brazil; Laboratório deBiologia de Insetos, Universidade Federal Fluminense, Niterói, RJ Brazil; Departamento de Entomologia Molecular, Instituto Nacional de Entomologia Molecular (INCT-EM), Rio de Janeiro, RJ Brazil; Laboratório de Biologia Molecular e Doenças Endêmicas, Instituto Oswaldo Cruz, Fundação Oswaldo Cruz (IOC/FIOCRUZ), Rio de Janeiro, RJ Brazil

**Keywords:** *Rhodnius prolixus*, *Trypanosoma cruzi*, Immune system, Antimicrobial peptides, Antibacterial activity, Microbiota

## Abstract

**Background:**

*Rhodnius prolixus* is a major vector of *Trypanosoma cruzi*, the causative agent of Chagas disease in Latin America. In natural habitats, these insects are in contact with a variety of bacteria, fungi, virus and parasites that they acquire from both their environments and the blood of their hosts. Microorganism ingestion may trigger the synthesis of humoral immune factors, including antimicrobial peptides (AMPs). The objective of this study was to compare the expression levels of AMPs (defensins and prolixicin) in the different midgut compartments and the fat body of *R. prolixus* infected with different *T. cruzi* strains. The *T. cruzi* Dm 28c clone (TcI) successfully develops whereas Y strain (TcII) does not complete its life- cycle in *R. prolixus*. The relative AMP gene expressions were evaluated in the insect midgut and fat body infected on different days with the *T. cruzi* Dm 28c clone and the Y strain. The influence of the antibacterial activity on the intestinal microbiota was taken into account.

**Methods:**

The presence of *T. cruzi* in the midgut of *R. prolixus* was analysed by optical microscope. The relative expression of the antimicrobial peptides encoding genes *defensin* (*defA*, *defB*, *defC*) and *prolixicin* (*prol*) was quantified by RT-qPCR. The antimicrobial activity of the AMPs against *Staphylococcus aureus*, *Escherichia coli* and *Serratia marcescens* were evaluated in vitro using turbidimetric tests with haemolymph, anterior and posterior midgut samples. Midgut bacteria were quantified using colony forming unit (CFU) assays and real time quantitative polymerase chain reaction (RT-qPCR).

**Results:**

Our results showed that the infection of *R. prolixus* by the two different *T. cruzi* strains exhibited different temporal AMP induction profiles in the anterior and posterior midgut. Insects infected with *T. cruzi* Dm 28c exhibited an increase in *defC* and *prol* transcripts and a simultaneous reduction in the midgut cultivable bacteria population, *Serratia marcescens* and *Rhodococcus rhodnii*. In contrast, the *T. cruzi* Y strain neither induced AMP gene expression in the gut nor reduced the number of colony formation units in the anterior midgut. Beside the induction of a local immune response in the midgut after feeding *R. prolixus* with *T. cruzi,* a simultaneous systemic response was also detected in the fat body.

**Conclusions:**

*R. prolixus* AMP gene expressions and the cultivable midgut bacterial microbiota were modulated in distinct patterns, which depend on the *T. cruzi* genotype used for infection.

**Electronic supplementary material:**

The online version of this article (doi:10.1186/s13071-016-1398-4) contains supplementary material, which is available to authorized users.

## Background

*Trypanosoma cruzi* is a protozoan parasite transmitted to vertebrate hosts by triatomine insects and is the causative agent of Chagas disease [1, 2]. This disease is a public health problem, and it is estimated that approximately 6 to 7 million people are infected with *T. cruzi* worldwide, mostly in Latin America [3]. *Rhodnius prolixus* has been considered one of the most efficient *T. cruzi* vector in South America [2, 4]. Consequently, the medical importance of this species has stimulated studies of its physiology, immunology and molecular biology, especially in experimental infections with *T. cruzi* [5–12]. Other important reasons to study *R. prolixus* include its rapid developmental cycle and ease of colonization in laboratories compared to other triatomine species [13].

*T. cruzi* exhibits a variety of genotypes with a wide range of heterogeneous populations that circulate through vertebrate and invertebrate hosts [14, 15]. Many morphological, physiological and ecological variations of this parasite, including its infectivity and pathogenicity [16–18], may explain the various clinical manifestations of Chagas disease observed in different geographic regions [19]. Currently, the intraspecific nomenclature of *T. cruzi* is based on grouping populations into six discrete typing units (DTUs) from TcI to TcVI [20].

The various *T. cruzi* genotypes differ in their success at developing inside the digestive tract of different triatomine species [21, 22]. Previous studies have demonstrated that the *T. cruzi* Y strain, classified as TcII, cannot colonize the gut of *R. prolixus*, while the *T. cruzi* Dm 28c clone, classified as TcI, successfully infects *R. prolixus* [20, 23, 24]. Many factors intrinsic to the invertebrate host have been linked to parasite development, including the activation of humoral immune responses and the influence of natural gut bacterial microbiota [22, 23, 25].

Humoral immunity in insects is composed of a number of effector molecules that are rapidly synthesized after microorganism invasion. One important humoral response is the production of inducible antimicrobial peptides (AMPs) [26, 27]. AMPs are mainly synthesized by fat body cells and can diffuse into the haemolymph, which circulates around the entire insect body. Consequently, AMPs are able to control infection [28, 29]. AMPs are also produced in other insect tissues such as gut epithelial cells, where parasites might interact directly and induce the local synthesis and release of these molecules [30].

Similar to most animals, insects contain a rich natural gut microbiota, which is essential for diverse functions in the host such as digestion and vitamin production [30, 31]. These observations raise the important question of how insects manage AMP synthesis after parasite infection while maintaining the intestinal bacterial microbiota population. A previous study of the *R. prolixus - T. cruzi* interaction revealed that the infective *T. cruzi* Dm 28c clone causes a decrease in the cultivable gut bacteria, unlike the non-infective *T. cruzi* Y strain [10]. Moreover, differential *T. cruzi* susceptibility to lytic activity from the bacteria *Serratia marcescens*, which is commonly present in the midgut of *R. prolixus*, has been observed [23, 32]. These results suggest that the success of *T. cruzi* colonisation in the *R. prolixus* midgut depends on the parasite DTU and its capacity to interact with the natural vector microbiota. The aim of this study was to investigate the influence of *T. cruzi* infection on the spatial and temporal expression of antimicrobial peptides in *R. prolixus* and gut microbiota.

## Methods

### *Rhodnius prolixus* maintenance and ethics statement

*R. prolixus* were maintained in a colony at *Laboratório de Bioquímica e Fisiologia de Insetos*, *Instituto Oswaldo Cruz*, under controlled temperature and humidity. The insects were fed defibrinated rabbit blood provided by the *Centro de Criação de Animais de Laboratório* (Cecal) in an artificial apparatus [13]. The rabbit blood was obtained according to the Ethical Principles in Animal Experimentation approved by the *Comissão de Ética no Uso de Animais do Instituto Oswaldo Cruz* (CEUA/IOC) under the protocol number L-0061/08 developed by *Conselho Nacional de Experimentação Animal/Ministério de Ciência e Tecnologia* CONCEA/MCT [33].

### *Trypanosoma cruzi* culture

The *T. cruzi* Dm 28c clone [34] and *T. cruzi* Y strain [35], previously classified as TcI and TcII, respectively [20], were maintained as epimastigote forms at 28 °C in brain heart infusion (BHI) media (Sigma-Aldrich) containing hemin and supplemented with 10 % heat-inactivated bovine foetal serum [13]. For insect infection, the parasites were used in the exponential growth phase. The number of parasites was quantified in a Neubauer chamber using an optical microscope.

#### Bacteria cultures

*Escherichia coli* K12 4401 and *Staphylococcus aureus* 9518 were obtained from the National Collection of Industrial and Marine Bacteria (NCIMB), Aberdeen, UK. *Serratia marcescens* RPH was previously isolated from *R. prolixus* and maintained at *Laboratório de Bioquímica e Fisiologia de Insetos*. All bacteria were kept at−70 °C in tryptone agar and 10 % glycerol.

### *Rhodnius prolixus* oral infection

Fifth instar nymphs were randomly chosen and fed with defibrinated rabbit blood containing *T. cruzi* epimastigotes of the Dm 28c clone or Y strain. The blood complement system was previously heat- inactivated by centrifugation at 1890 × *g* for 15 min at 4 °C and incubation of the plasma (supernatant) for 30 min at 55 °C. Subsequently, the plasma was mixed with phosphate buffered saline (PBS)-washed erythrocytes, and the parasites were added to the reconstituted blood at a final concentration of 1 × 10^7^ epimastigotes/mL. Additionally, groups of insects received a blood meal containing a combination of two antibiotics (penicillin and ampicillin) with or without *T. cruzi* Dm 28c epimastigotes. Each antibiotic was administrated in a final concentration of 300 μg/ml of blood. Uninfected control insects were fed on inactivated blood without parasites. Only fully engorged fifth instar *R. prolixus* nymphs were used for the experiments.

### *T. cruzi* quantification in the *R. prolixus* midgut

The entire digestive tract was dissected and individually homogenized in 1.0 mL PBS. The parasites were counted using a Neubauer haemocytometer as previously described [36].

#### Quantification of the *R. prolixus* bacterial midgut microbiota (CFU)

The anterior and posterior midgut were separately dissected from fifth instar nymphs infected or uninfected with *T. cruzi* (*n* = 9). The cultivable microbiota population was quantified by counting the colony forming units (CFU) 7 days after feeding (DAF) as previously described [10]. Briefly, the midgut samples were serially tenfold diluted with sterile PBS, and 20 μL aliquots of each dilution were spread on a Petri dish in sterile BHI agar (Sigma-Aldrich) culture medium. The plates were incubated at 30 °C for 24 h, and the CFUs were quantified. As control, PBS was plated to check the sterility of the experiments. Additionally, RT-qPCR was performed using cDNA from 3 pools of 10 anterior midguts from different insect groups: control uninfected insects, treated with antibiotics (as described above), infected with *T. cruzi* Dm 28c; treated with antibiotics simultaneously infected with *T. cruzi* Dm 28c epimastigotes.

### Haemolymph and midgut antibacterial assays

Anterior and posterior midguts were collected from dissected insects (nine pools of three insects each) and prepared as previously described [12, 36]. Haemolymph samples were pooled 5 DAF from 10 insects from three different experiments and diluted 1:1 in ultrapure water in sterile 1.5 ml tubes containing a few crystals of phenylthiourea to avoid melanisation. The bacteria (*S. aureus*, *E. coli* and *S. marcescens*) were grown as previously described [36]. The antibacterial activity of the insect samples was assessed by modified turbidimetric assays (TB) [10]. All experiments were carried out at least in triplicate (*n* = 9). The detected antibacterial activities represent the *R. prolixus* inducible humoral immune molecules and other factors produced by midgut microbiota.

### Quantification of antimicrobial peptide gene expression and intestinal bacteria by RT-qPCR

Insects at 1 and 7 DAF (*T. cruzi* infected and non-infected) were dissected to prepare three pools each of five anterior midguts, posterior midguts and fat body as previously described [12]. Total RNA was extracted using a NucleoSpin® RNA II Kit (Macherey-Nagel, Düren, Germany) following the manufacturer’s instructions and quantified using a NanoDrop 2000 Spectrophotometer (Thermo Scientific, Waltham, MA, USA). Synthesis of cDNA was carried out with a First-Strand cDNA Synthesis Kit (GE Healthcare, Buckinghamshire, UK) following the manufacturer’s protocol using 2.5 μg of total RNA and the pd(N)_6_ primer. cDNA was quantified by fluorescence using a Qubit Fluorimeter (Life Technologies) with the ssDNA assay kit. Real-time quantitative polymerase chain reactions (RT-qPCR) were conducted using an ABIPRISM 7500 Sequence Detection System (Applied Biosystems) at the PDTIS/FIOCRUZ facilities (Real-Time PCR Platform RPT-09A). The present study analysed the gene expression of three *R. prolixus* defensins (*defA*, *defB* and *defC*) and prolixicin (*prol*). The specific primers for the AMP genes, *R. prolixus* reference genes and 16S rRNA primers for relative quantification of *S. marcescens* and *R. rhodnii* were used as previously published or were designed based on the respective sequence (Additional file 1) [9, 37, 38], using the expression of control uninfected insects as calibrators. Each reaction was run in duplicate for each pool of insects (*n* = 3). Each well contained 10 ng cDNA, primer pairs (0.25 μM) and the qPCR master mix DyNAmo ColorFlash SYBR Green qPCR Kit (Thermo Fisher Scientific) at a final volume of 20 μl. The cDNA was amplified at 95 °C for 10 min followed by 40 cycles of 95°for 15 s and 60 °C for 1 min. As negative controls, PCR reactions were carried out without cDNA template to assess primer dimer formation or contamination in the reactions. A melting curve analysis was carried out to confirm that only a single product was amplified for each target. The AMP genes in the tissues of *R. prolixus* infected with *T. cruzi* were quantified by the comparative Ct (∆∆Ct) method [39] normalized with the *R. prolixus* reference genes *α-tubulin* and *GAPDH*. Data were analysed by the Expression Suite v1.0.3 software (Life Technologies), considering the amplification efficiency of each target.

### Statistical analysis

All obtained data were analyzed using Student’s *T*-test and 1-way ANOVA and the GraphPad Prism 5 software. Significance levels are shown in the respective figures and legends, which were considered statistically different when *p* < 0.05.

## Results

### Quantification of *T. cruzi* in the *R. prolixus* midgut

The parasite populations in the entire midgut of the 5^th^ instar *R. prolixus* nymphs were quantified from 2 to 7 days after feeding (DAF) with blood containing parasites. Two DAF, the average concentration of *T. cruzi* Dm 28c (75 × 10^4^ parasites/mL) was ten times higher than that of *T. cruzi* Y (7.5 × 10^4^ parasites/mL) in the insect gut (Additional file 2, *p* < 0.001). On the 5^th^ DAF, a decrease in the number of parasites from both the *T. cruzi* strains was detected, although the *T. cruzi* Dm 28c number was two times greater than *T. cruzi* Y (Additional file 2, *p* < 0.01). Moreover, no *T. cruzi* Y strain parasites were detected in the *R. prolixus* gut samples analysed on the 7^th^ DAF, while on the same day the *T. cruzi* Dm 28c population increased in all insect guts analysed (Additional file 2).

### Analysis of the *R. prolixus* midgut microbiota-colony forming units (CFU) and RT-qPCR

**Fig. 1:**
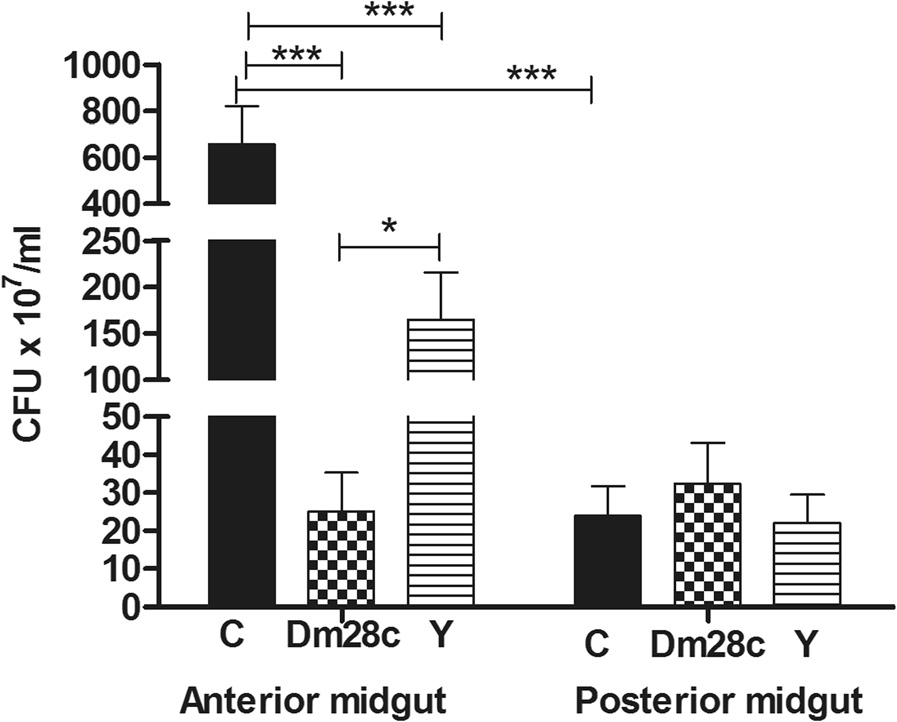
Bacteria populations in the *Rhodnius prolixus* midgut infected with *Trypanosoma cruzi.* Colony forming units (CFU) were counted in the anterior and posterior midgut samples seven days after feeding the insects with blood at a final concentration of 1 × 10^7^ epimastigotes/mL. Insect treatments: black columns-control insects (C); grid columns-*T. cruzi* Dm 28c clone-infected insects (Dm 28c); striped columns-*T. cruzi* Y strain-infected insects (Y). Bars represent the mean ± SEM of three independent experiments with nine pools of insects (*n* = 9). Means were compared using Student’s *T*-test or Mann—Whitney test; *** *p* < 0.001, * *p* < 0.05

The cultivable bacterial microbiota population in the 5^th^ instar nymphs infected with *T. cruzi* was evaluated separately for the anterior and posterior midgut using CFU counts. Infection with both *T. cruzi* strains significantly reduced the bacterial population in the *R. prolixus* anterior midgut on the 7^th^ DAF. However, *T. cruzi* Dm 28c caused a stronger CFU reduction (*p* < 0.001) (2.5 × 10^8^ CFU/mL−26-fold less) than *T. cruzi* Y (*p* < 0.05) (1.64 × 10^9^ CFU/mL−4-fold less) in comparison with uninfected insects (6.57 × 10^9^ CFU/mL) (Fig. 1). *T. cruzi* infection did not significantly alter the CFU counts in the posterior midgut compared with control insects.

After *T. cruzi* Dm 28c infection a 2.6-fold reduction of the *S. marcescens* bacterial number was detected (Additional file 3A, *p* < 0.01) and the *R. rhodnii* load decreased 3.5-fold (Additional file 3B, *p* < 0.01) in the anterior midgut. In insects treated with antibiotics, *S. marcescens* was reduced 10-fold (Additional file 3A, *p* < 0.001) whereas the *R. rhodnii* population increased 3-fold (Additional file 3B, *p* < 0.001). Data were always compared to control insects fed only on blood.

### Haemolymph and midgut antibacterial detection

**Fig. 2:**
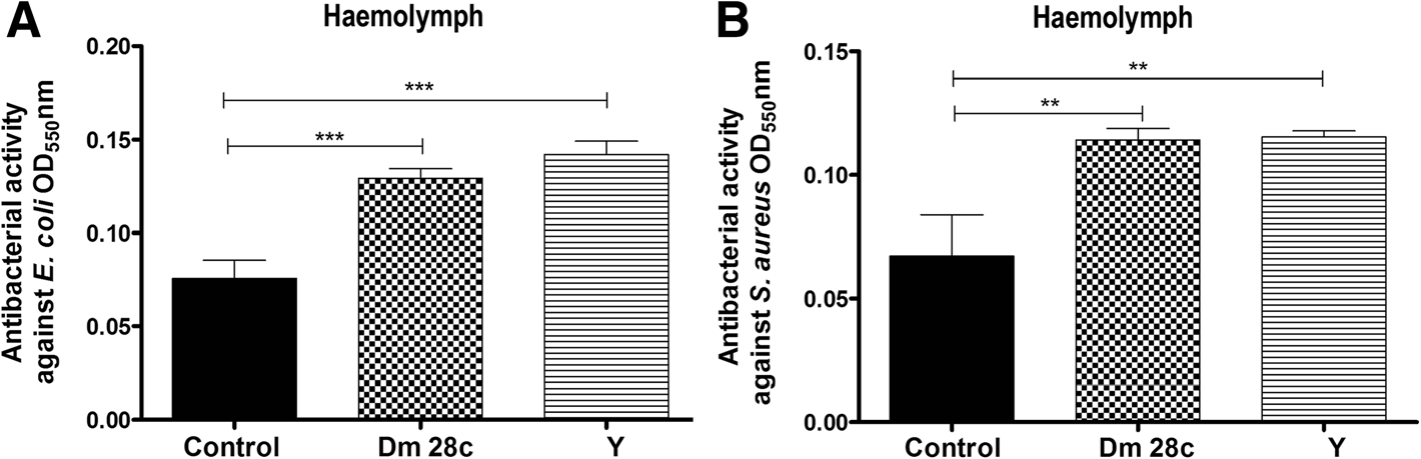
Antibacterial activities in haemolymph samples of *Rhodnius prolixus* infected with *Trypanosoma cruzi. R. prolixus* 5^th^ instar nymphs were fed inactivated blood containing the *T. cruzi* Dm 28c clone or Y strain at a final concentration of 1 × 10^7^ epimastigotes/mL. The antibacterial activities were assayed with haemolymph collected five days after infection and tested against (**a**) *E. coli* or (**b**) *S. aureus*. Haemolymph samples were prepared from: black columns-control, uninfected insects; grid columns-infected with *T. cruzi* Dm 28c; striped columns-infected with *T. cruzi* Y strain. Antibacterial activity was measured through optical densities using the turbidimetric assay (OD_550_ nm) after 19 h of incubation of haemolymph samples with bacteria. Bars represent the mean ± SEM of three independent experiments with nine pools of insects (*n* = 9). Means were compared using one-way ANOVA and Student’s *T*-test; *** *p* < 0.001, ** *p* < 0.01

The *R. prolixus* haemolymph antibacterial activity was demonstrated using TB assays. The haemolymph of uninfected control insects demonstrated antibacterial activities that inhibited the growth of both Gram-negative and Gram-positive bacteria (Fig. 2). However, haemolymph from both the *T. cruzi* infected insect groups exhibited significantly higher antibacterial activity than the haemolymph samples from the control insects (Fig. 2a, *p* < 0.001; Fig. 2b, *p* < 0.001).

**Fig. 3:**
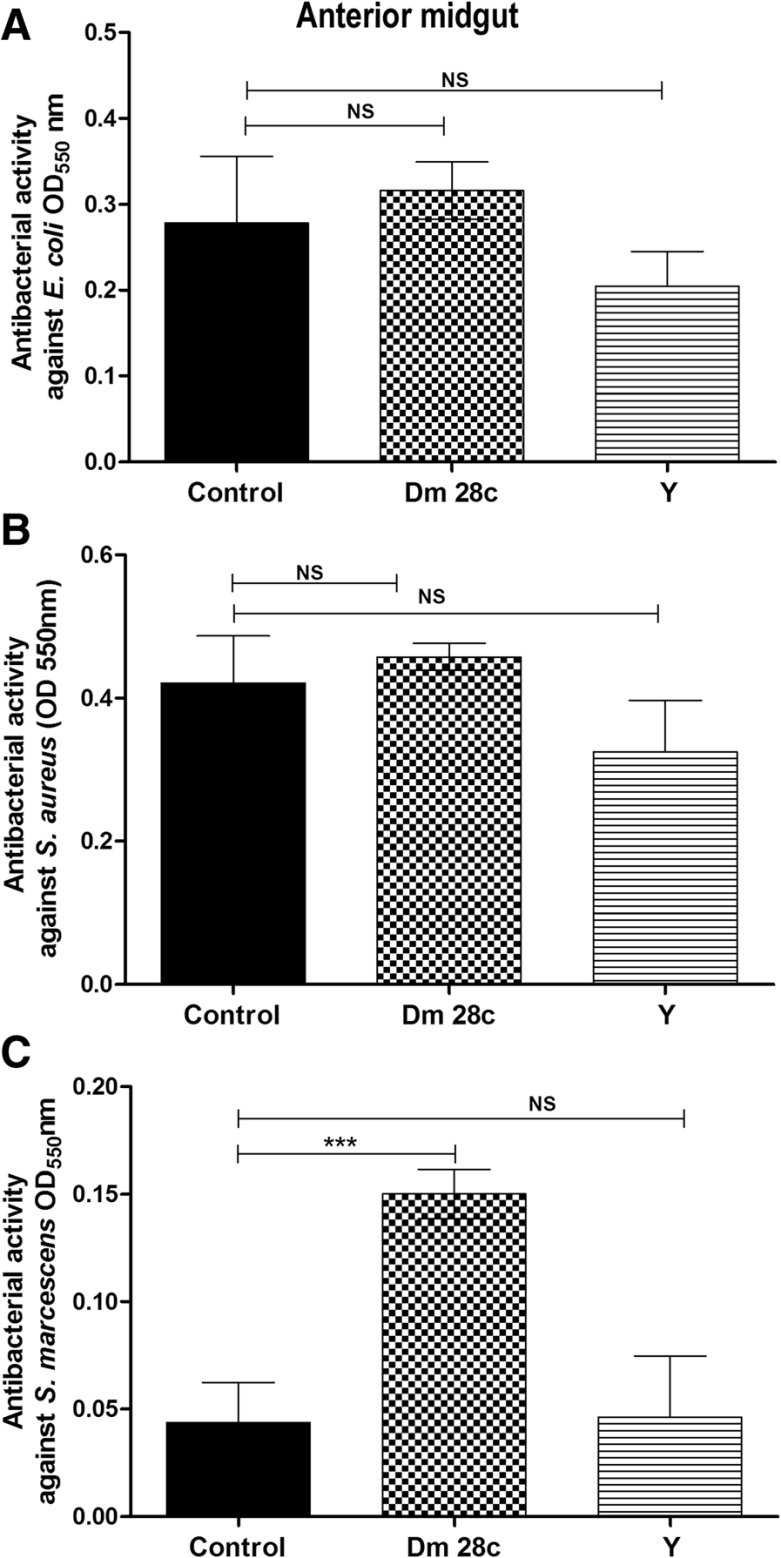
Antibacterial activity in the anterior midgut of *Rhodnius prolixus* infected with *Trypanosoma cruzi. R. prolixus* 5^th^ instar nymphs were fed inactivated blood containing the *T. cruzi* Dm 28c clone or Y strain at a final concentration of 1 × 10^7^ epimastigotes/mL. The antibacterial activities were measured in vitro in anterior midgut samples seven days after infection and tested against (**a**) *E. coli*, (**b**) *S. aureus* and (**c**) *S. marcescens*. Treatments: black columns-uninfected insects; grid columns-*T. cruzi* Dm 28c-infected insects; striped columns-*T. cruzi* Y-infected insects. Antibacterial activity was measured through optical densities using the turbidimetric assay (OD_550_ nm) after 19 h of incubation of anterior midgut samples with bacteria. Bars represent the mean ± SEM of three independent experiments with nine pools of insects (*n* = 9). Means were compared using one-way ANOVA and Student’s *T*-test; *** *p* < 0. 001, NS = not significant

The optical densities of the *R. prolixus* anterior midgut incubated with different bacteria showed that only the *T. cruzi* Dm 28c infection significantly increased the antibacterial activity against *S. marcescens* (Fig. 3c, *p* < 0.001). The activities measured against *E. coli* and *S. aureus* in insects infected with both *T. cruzi* strains were not statistically significant compared to the control insects (Fig. 3a, 3b).

**Fig. 4:**
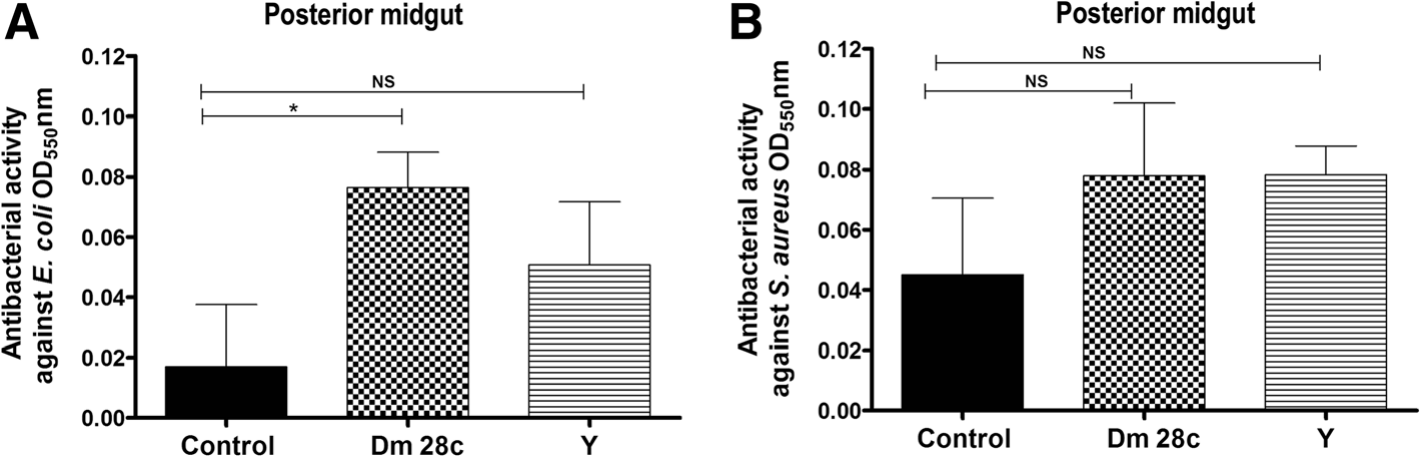
Antibacterial activity in the posterior midgut of *Rhodnius prolixus* infected with *Trypanosoma cruzi. R. prolixus* 5^th^ instar nymphs were fed inactivated blood containing the *T. cruzi* Dm 28c clone or Y strain at a final concentration of 1 × 10^7^ epimastigotes/mL. The antibacterial activities were measured in vitro in posterior midgut samples seven days after infection and tested against (**a**) *E. coli* and (**b**) *S. aureus*. Treatments: black columns-uninfected insects; grid columns-*T. cruzi* Dm 28c-infected insects; striped columns-*T. cruzi* Y-infected insects. Antibacterial activity was measured through optical densities using the turbidimetric assay (OD_550_ nm) after 19 h of incubation of posterior midgut samples with bacteria. Bars represent the mean ± SEM of three independent experiments with nine pools of insects (*n* = 9). Means were compared using one-way ANOVA and Student’s *T*-test; * *p* < 0.05

Antibacterial assays of the *R. prolixus* posterior midgut samples demonstrated that only *T. cruzi* Dm 28c infection induced an increase in the antibacterial activity against *E. coli* (Fig. 4a, *p* < 0.01). No differences were observed for the activities against *S. aureus* in insects infected with the two *T. cruzi* strains compared to the control insects in this same midgut compartment (Fig. 4b).

### Quantification of antimicrobial peptide gene expression (RT-qPCR)

**Fig. 5:**
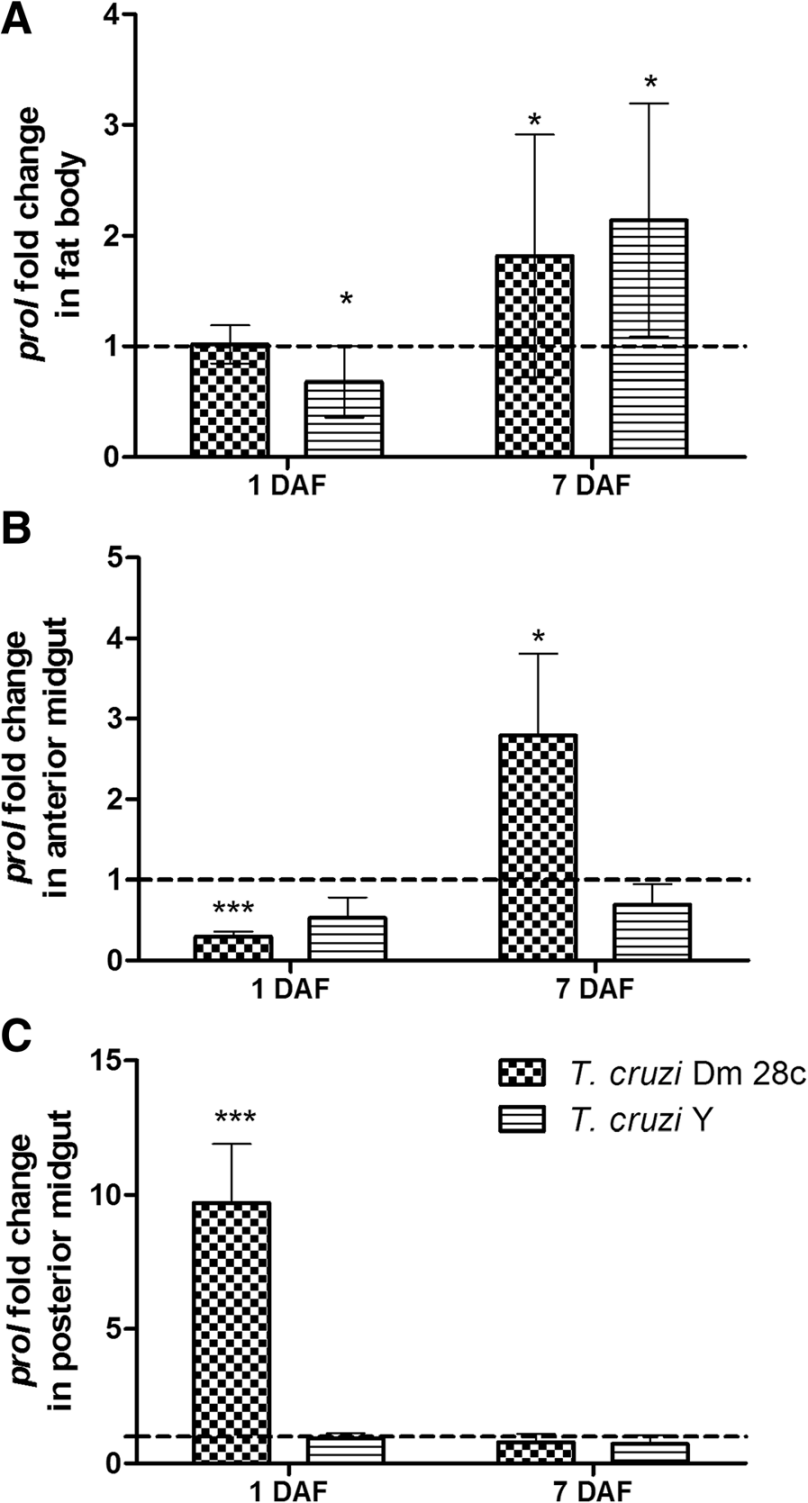
Spatial and temporal prolixicin relative gene expression in *Rhodnius prolixus* orally infected with *Trypanosoma cruzi. R. prolixus* 5^th^ instar nymphs were fed inactivated blood containing the *T. cruzi* Dm 28c clone or Y strain at a final concentration of 1 × 10^7^ epimastigotes/mL. Data were quantified using the gene expression of uninfected insects as the calibrator represented by the dotted horizontal line on each graph and shown as the relative *prol* expression in the (**a**) fat body, (**b**) anterior midgut and (**c**) posterior midgut on the 1^st^ and 7^th^ days after feeding (DAF). Treatments: grid columns-relative *prol* expression in insects infected with *T. cruzi* Dm 28c; striped columns-relative *prol* expression in insects infected with *T. cruzi* Y. Bars represent the mean ± SEM of two independent experiments with six pools of insects (*n* = 6). Means were compared using one-way ANOVA and Student’s *T*-test; *** *p* < 0.001, * *p* < 0.05

Modulations in the expression of AMP genes in the *R. prolixus* 5^th^ instar nymphs infected with *T. cruzi* Dm 28c and *T. cruzi* Y strain were verified in different tissues at 1 and 7 DAF. All data obtained here were compared to the gene expression in control insects, which was given the value 1.0 and is represented in the figures as horizontal dotted lines along the Y-axis. Expression of *prol* in both the *T. cruzi* Dm 28c and Y strains was significantly upregulated at the transcript level in the fat body on the 7^th^ DAF in comparison to control insects (Fig. 5a, *p* < 0.05). However, only the *T. cruzi* Dm 28c infection modulated *prol* expression in the *R. prolixus* midgut, with distinct patterns in the different compartments. In the anterior midgut, *prol* transcript levels were significantly lower in infected insects than in controls on the 1^st^ DAF (Fig. 5a, *p* < 0.001). On the 7^th^ DAF, the *prol* transcript levels had increased by 2.5-fold in this same midgut compartment of *T. cruzi* Dm 28c-infected insects (Fig. 5b, *p* < 0.05). However, the opposite pattern was observed in the posterior midgut, where the *prol* expression was 10-fold higher in the *T. cruzi* Dm 28c-infected insects on the 1^st^ DAF (Fig. 5c, *p* < 0.001), while on the 7^th^ DAF the *prol* transcript levels decreased, reaching the same levels as in the control insects (Fig. 5c). Additionally, the *T. cruzi* Y strain-infected insects exhibited *prol* gene expression similar to control insects in both the anterior and posterior midgut on the 1^st^ and 7^th^ DAF (Fig. 5b, c).

**Fig. 6:**
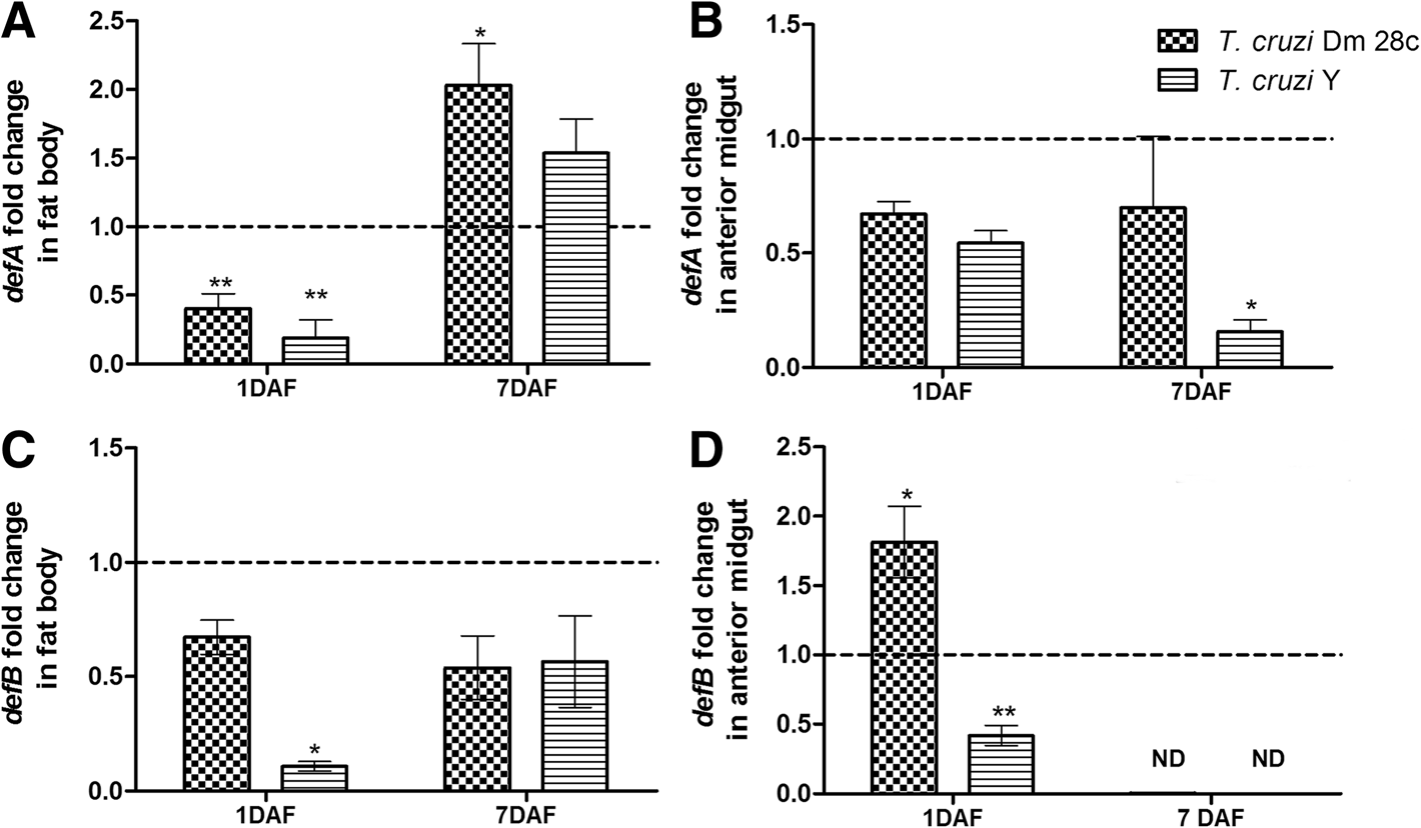
Spatial and temporal defensin A and B relative gene expression in *Rhodnius prolixus* orally infected with *Trypanosoma cruzi. R. prolixus* 5^th^ instar nymphs were fed inactivated blood containing the *T. cruzi* Dm 28c clone or Y strain at a final concentration of 1 × 10^7^ epimastigotes/mL. Data were quantified using the gene expression of uninfected insects as the calibrator, represented by the dotted horizontal line on each graph, and shown as the relative expression of (**a**) *defA* in the fat body, (**b**) *defA* in the anterior midgut, (**c**) *defB* in the fat body and (**d**) *defB* in the anterior midgut on the 1^st^ and 7^th^ days after feeding (DAF). Treatments: grid columns-relative gene expression in insects infected with *T. cruzi* Dm 28c; striped columns-relative gene expression in insects infected with *T. cruzi* Y. Bars represent the mean ± SEM of two independent experiments with six pools of insects (*n* = 6). Means were compared using one-way ANOVA and Student’s *T*-test; ** *p* < 0.01, * *p* < 0.05, ND = not determined

Infection of *R. prolixus* with both the *T. cruzi* Dm 28c and Y strains down regulated the *defA* levels on the 1^st^ DAF in the fat body (Fig. 6a, *p* < 0.01). On the 7^th^ DAF, a significant 2-fold increase was detected in the *defA* levels in the *T. cruzi* Dm 28c-infected insects (Fig. 6a, *p* < 0.05), while the *T. cruzi* Y-infected insects presented the same gene levels as the control (Fig. 6a). In the anterior midgut, only the infection with *T. cruzi* Y modulated the *defA* transcript levels, which were significantly lower than in the control insects (Fig. 6b, *p* < 0.05).

The ingestion of *T. cruzi* Y down regulated the *defB* levels on the 1^st^ DAF in the *R. prolixus* fat body and midgut (Fig. 6c, *p* < 0.05; Fig. 6d, *p* < 0.01). However, *T. cruzi* Dm 28c significantly upregulated *defB* levels in the anterior midgut, but only on the 1^st^ DAF (Fig. 6d, *p* < 0.05). No *defB* transcripts were detected in the anterior midgut on the 7^th^ DAF for either of the *T. cruzi* strains (Fig. 6d).

**Fig. 7:**
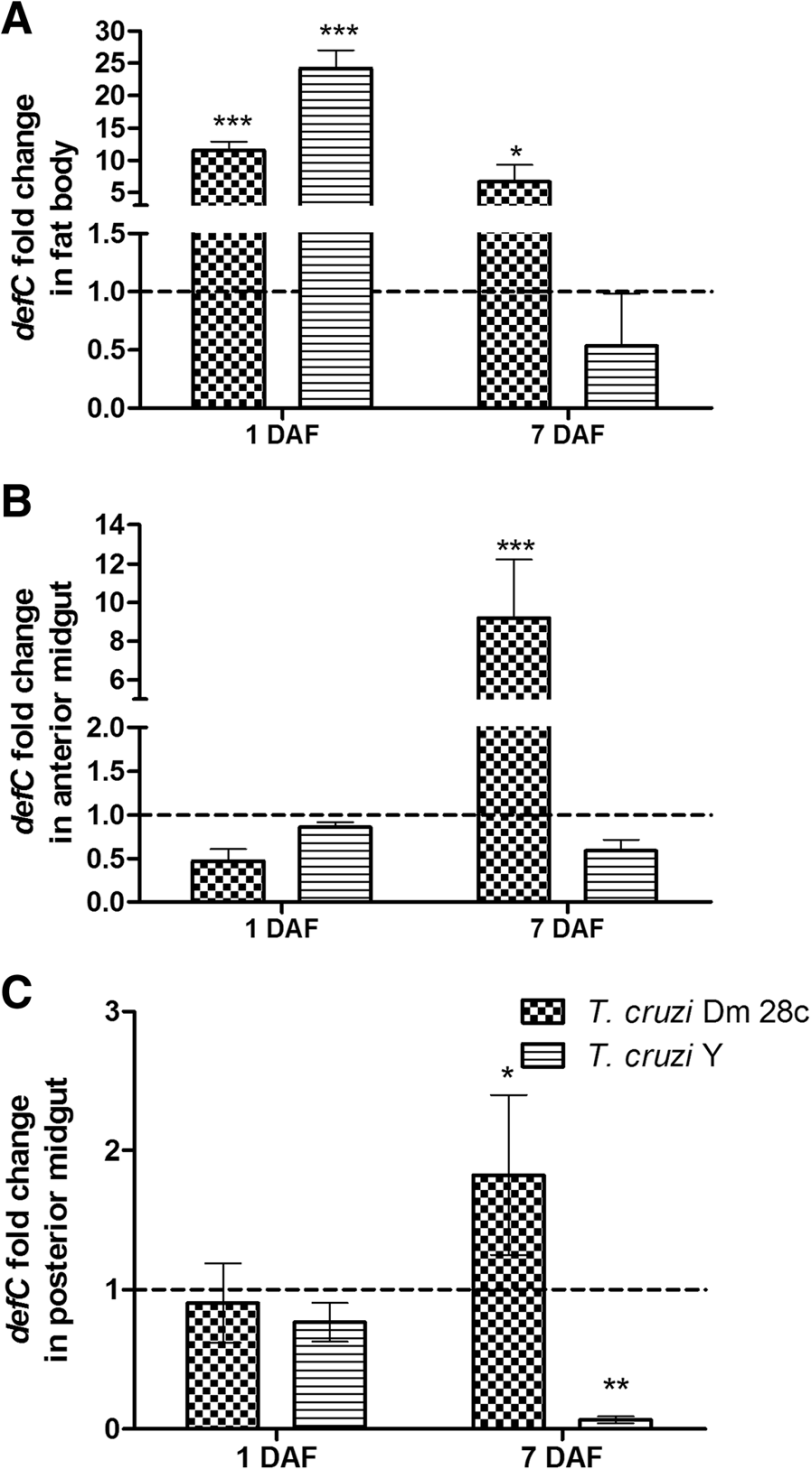
Spatial and temporal defensin C relative gene expression in *Rhodnius prolixus* orally infected with *Trypanosoma cruzi. R. prolixus* 5^th^ instar nymphs were fed inactivated blood containing the *T. cruzi* Dm 28c clone or Y strain at a final concentration of 1 × 10^7^ epimastigotes/mL. Data were quantified using the gene expression of uninfected insects as the calibrator, represented by the dotted horizontal line on each graph, and shown as the *defC* relative expression in the (**a**) fat body, (**b**) anterior midgut and (**c**) posterior midgut on the 1^st^ and 7^th^ days after feeding (DAF). Treatments: grid columns-*defC* relative expression in insects infected with *T. cruzi* Dm 28c; striped columns-*defC* relative expression in insects infected with *T. cruzi* Y. Bars represent the mean ± SEM of two independent experiments with six pools of insects (*n* = 6). Means were compared using one-way ANOVA and Student’s *T*-test; *** *p* < 0.001, ** *p* <0.01, * *p* < 0.05

Both *T. cruzi* strains strongly induced *defC* expression in the insect fat body on the 1^st^ DAF. While *T. cruzi* Dm 28c increased *defC* levels 11-fold (Fig. 7a, *p* < 0.001), *T. cruzi* Y increased *defC* 24-fold (Fig. 7a, *p* < 0.001) compared with non-infected insects. In the anterior and posterior midgut, only *T. cruzi* Dm 28c significantly upregulated *defC* levels on the 7^th^ DAF (Fig. 7b, *p* < 0.001; Fig. 7c, *p* < 0.05), whereas *T. cruzi* Y down regulated these genes in the posterior midgut on the 7^th^DAF (Fig. 7c, *p* < 0.01).

In insects fed on blood containing antibiotics *defC* expression decreased 1.72-fold (Additional file 4, *p* < 0.05) in the anterior midgut when compared to control non-infected insects. However, in insects treated with antibiotics and infected with *T. cruzi* Dm 28c, *defC* expression was 4-fold higher (Additional file 4, *p* < 0.01) in comparison to *T. cruzi* infected insects without antibiotics treatment and 14-fold higher than control, uninfected (Additional file 4, *p* < 0.001). Summary of all results obtained are shown in Additional files 5 and 6.

## Discussion

This study attempted to understand the influence of *T. cruzi* Dm 28c and *T. cruzi* Y infections on the modulation of AMP gene expression and antibacterial activity in different *R. prolixus* tissues, thereby altering the gut bacterial microbiota and parasite survival. It has already been demonstrated that *T. cruzi* Y strain is rapidly lysed, while *T. cruzi* Dm 28c complete its life-cycle in the gut of *R. prolixus* [10, 23]. Comparing the infection of these two *T. cruzi* genotypes in *R. prolixus*, we found that Dm 28c induced:(i) a reduction of the CFU bacterial number, (ii) an  increase of antibacterial activity against *S. marcescens*, (iii) an enhancement of *prol* and *defC* expression, and (iv) a decrease of *S. marcescens* and *R. rhodnii* load in the anterior midgut. In the present study, the insects fed on blood containing both antibiotics and *T. cruzi* Dm 28c, *defC* expression increased 4-fold in comparison to infected insects lacking antibiotics. In this context, a previous study demonstrated that *R. prolixus* fed on blood containing *T. cruzi* Dm 28c and antibiotics presented significantly increased parasite numbers and a reduced cultivable bacterial population in the midgut [10]. Together, all these findings suggest a regulatory function of defensin C on *R. prolixus* microbiota, promoting the reduction of intestinal bacteria number and subsequently the parasite development. *S. marcescens - *one cultivable bacteria commonly found in the triatomine’s gut- has been associated with trypanolytic effects [23, 36, 40, 41]. This cytotoxic bacterium is a natural barrier that influences the establishment of parasites [6, 10, 23]. In contrast to Dm 28c, *T. cruzi* Y strain is apparently not able to stimulate the local immune response and overcome this barrier.

Antimicrobial effects of recombinant AMP prolixicin have been assayed against different Gram-negative bacterial species, showing higher activity against *E. coli* than *S. marcescens* [9]. Here, the synergistic effects of prolixicin associated with the effects of defensin C could explain the increased antibacterial activity against *S. marcescens* and the decrease of *R. rhodnii* and *S. marcescens* bacterial load in the anterior midgut after *T. cruzi* Dm 28c infection. On the other hand, *R. prolixus* infected with the *T. cruzi* strain Chile 5 did not exhibit a reduced *R. rhodnii* population [42]. These results suggest that the capacity of *T. cruzi* to colonize the *R. prolixus* midgut might depend on its genotypic characteristics combined with its ability to modulate (directly or indirectly) the host natural microbiota. In a recent study, the silencing of *rpRelish* (Nf-κB transcription factor of AMPs in the IMD pathway) resulted in an increase of *R. rhodnii* CFU, which did not affect *T. cruzi* Dm 28c clone development in *R. prolixus* midgut [43].

Many studies have described how the intestinal microbiota of insect vectors affect the life-cycle of parasites, including nutrient competition between these microorganisms [44, 45]. Native bacteria in the *Anopheles* midgut negatively affect certain species of *Plasmodium* by direct contact between the microorganisms involved and by the induction of the immune response mediated by commensal bacteria [46–48]. In contrast, parasitic infections capable of modulating immune peptide synthesis in insect hosts [36, 49, 50] might interfere with the growth of certain bacteria species of the microbiota, as seems to occur in *Trypanosoma rangeli* infection in *R. prolixus* [36]. Moreover, serine protease inhibitors from the Kazal family in the anterior midgut of *T. cruzi*-infected *R. prolixus* were recently shown to be involved in the modulation of the intestinal microbiota [51].

The presence of bacteria or parasites in the digestive tract stimulates the systemic secretion of AMPs into the haemolymph of different insects, including species of *Phlebotomus*, *Glossina* and *Drosophila*, even without the invasion of these microorganisms into the haemocoel [49, 52–54]. After induction of AMP gene expression in the fat body, the respective peptides are initially secreted directly into the haemolymph. Later, the AMPs can diffuse throughout the insect body, representing a systemic response to oral parasite infection [29]. The induction of AMP expression in the fat body by parasites confined to the midgut could be due to immune signalling by molecules such as nitric oxide (NO), representing a host anticipation strategy to prevent a widespread infection [54, 55]. A similar pattern was observed in this study. The oral Infection of *R. prolixus* by both *T. cruzi* genotypes triggered a rapid increase of the *defC* gene transcriptional levels in the fat body. High levels of mature DefC in the fat body might be directly related to the increased antibacterial activity detected in vitro in the haemolymph.

During *T. cruzi* Dm 28c development in *R. prolixus,* higher numbers of epimastigotes are found in the anterior midgut until the fifth day after infection. Thereafter, they tend to inhabit the posterior midgut [56]. One day after *T. cruzi* Dm 28c infection, reduced *prol* levels were found in the anterior midgut, while *prol* levels had increased in posterior midgut. The *T. cruzi* Y infection was not capable of upregulating the expression of the analysed AMPs in the *R. prolixus* midgut. The expression of AMPs in intestinal epithelial cells is considered a local immune response activated by the direct contact of the parasites with the insect tissue [29, 57]. Here, the systemic response of *R. prolixus* to the different parasite strains was similar, although local responses to TcI and TcII infection exhibited differing profiles.

The parasite *P. berghei* modulates defensin gene expression in the gut and salivary gland epithelium of *Anopheles gambiae*, tissues where the parasite is found during its life-cycle [58]. *Leishmania major* infection also induces defensin expression in the haemolymph and midgut of the insect *Phlebotomus duboscqi* [49], although infection by *Leishmania mexicana* totally abolished *def1* expression in *Lutzomyia longipalpis* [50]. Interestingly, the infection of *R. prolixus* with the *T. rangeli* Macias strain also upregulated *defC* levels in the midgut regions where the parasite was found [36]. In the present study, the presence of *T. cruzi* Dm 28c in different midgut regions had a similar effect, enhancing *defC* expression. These results support the hypothesis that defensin genes are modulated by protozoan infections in insects and not directly by the CFU bacterial microbiota population.

Several defensin-encoding genes have been identified in different triatomine species [11, 37, 59–61]. After the analysis of the primary defensin structure and the transcript abundance of defensin-encoding genes in *T. brasiliensis*, it was proposed that these peptides have different biological targets [60]. In agreement, the three *R. prolixus* defensin encoding genes were differently expressed in response to the invading microorganism. In the present work, only *defC* was up regulated by *T. cruzi* Dm 28c, 7 DAF in the *R. prolixus* midgut. A similar result was also observed in *T. rangeli* infected insects [36]. On the other hand, *defA* and *defB* were upregulated in the midgut in response to Gram-positive bacterial oral infection [12].

Prolixicin was recently identified in *R. prolixus* haemolymph and shows structural similarities with another AMP family, the attacins [9]. The influence of attacins on the establishment of *Trypanosoma brucei* was previously demonstrated in the tsetse fly [62, 63]. *T. brucei* infection upregulated the attacin genes in the fat body and midgut of the insect vector. In addition, insects naturally resistant to *T. brucei* infection present high levels of attacin even in non-infected flies, suggesting that this AMP interferes with *T. brucei* development [62, 63]. Attacin and prolixicin possess high toxicity against the Gram-negative *E. coli* [9, 62], suggesting that the structural similarity between these AMPs may also extend to their microbial targets. Because prolixicin shares similar characteristics with attacins, this AMP may also have a toxic effect against trypanosomatids. The prolixicin gene expression seems to be downregulated according to the local concentration of *T. cruzi* epimastigotes, as observed in experimental infections with the *T. rangeli* Macias strain that successfully infects the *R. prolixus* midgut [36]. The results obtained from *T. cruzi* infection in *R. prolixus* suggest that suppression of *prol* genes may represent one survival mechanism of trypanosomatids in the insect gut.

The genetic variability of *T. cruzi* is expressed in different compositions of cell surface molecules, such as sugars and proteins [64]. *T. cruzi* populations belonging to the TcI genotype contain more galactose residues in their cell surfaces than the TcII populations [65]. Membrane proteins such as the virulence factor trans-sialidase are also differently distributed in TcI and TcII genotypes [66]. Other membrane-bound proteins of *T. cruzi*, such as mucins, interact by the adhesion of the flagellum to the epithelial cell and perimicrovillar membranes of the midgut, a crucial step for parasite development and infectivity [67]. The role of trans-sialidases and mucins as virulent factors in the recognition of *T. cruzi* and in the induction of specific immune responses has been demonstrated in vertebrate models [68–70]. Therefore, the distinct immune response results obtained in the present study from *R. prolixus* infection by the two *T. cruzi* strains might be an effect of differences on *T. cruzi* membrane structures. Because *R. prolixus* is predominantly infected with TcI [71, 72], this genotype may be better adapted to this triatomine species due to a modulation of the immune response favouring the establishment of the parasite.

Currently, efforts to control human diseases transmitted by insects are focused on vector control, specifically in the construction of genetically modified intestinal bacteria that control their natural parasites by recombinant expression of an AMP or by RNAi [73, 74]. Knowledge about how different *T. cruzi* genotypes modulate antimicrobial peptide expression in *R. prolixus* can assist in selecting the genes to be manipulated. RNAi technology is an interesting tool to verify in the future the effect of each AMP gene expression on the parasite development and on the gut microbiota composition of *R. prolixus*. It would also be important to perform studies using aposymbiotic insects - which might have an effect on the triatomine immunity - to clarify the specific effects of each bacteria species and *T. cruzi* infections. However, these conditions would be artificial as in nature insects are frequently found inhabiting a great variability of bacterial species in the intestine and are infected with *T. cruzi* simultaneously [41]. To further clarify the tripartite relation between parasites, microbiota and insects, the measurement of protein levels and proteomic analyses will be useful in the future.

## Conclusions

*T. cruzi* Dm 28c-infected *R. prolixus* showed increased *defC* and *prol* expression levels in the anterior midgut and a higher antibacterial activity against *S. marcescens* that could be related to the drastic reduction of the cultivable bacterial microbiota, *S. marcescens* and *R. rhodnii*. In contrast, these effects were not observed in insects infected with the *T. cruzi* Y strain, which might explain why this strain does not develop in *R. prolixus*.

## Additional files

Additional file 1: Oligonucleotide primers used for qPCR analysis. (DOC 46 kb) Additional file 2: Parasite population in *Rhodnius prolixus* digestive tract. The numbers of *T. cruzi* Dm 28c and Y strain parasites were estimated in the whole digestive tracts of *R. prolixus* 5^th^ instar nymphs at different days after feeding (DAF). Each point represents the number of parasites in an individual insect, and bars indicate the median. Means were compared using Student’s *T*-test or Mann-Whitney test; *** *p* < 0.001, * *p* < 0.05. (BMP 64 kb) Additional file 3: Determination of bacterial load in the anterior midgut of *Rhodnius prolixus* after antibiotic treatment and *Trypanosoma cruzi* Dm 28c infection by RT-qPCR. *R. prolixus* anterior midgut were analysed 7 days after feeding on inactivated blood containing antibiotics (ampicillin 300 μg/ml plus penicillin 300 μg/ml of blood) and *T. cruzi* Dm 28c clone at a final concentration of 1 × 10^7^ epimastigotes/mL. Relative expression of 16S-rRNAS of A−*Serratia marcescens* B−*Rhodococcus rhodnii*. Treatments: black column—control insects fed only on blood; white column-insects fed on blood containing antibiotics; grid column-insects fed on blood containing *T. cruzi*; striped columns-insects fed on blood containing antibiotics and *T. cruzi*. Two biological samples in triplicate were used for each group. All data were normalized to the *R. prolixus* α-tubulin, representing the mean of identical triplicates ± standard error. Bars represent the mean ± SEM of 3 independent experiments with 3 pools of insects (*n* = 3). Means were compared using Student’s *T*-test; *** *p* < 0.001, ** *p* < 0.01, * *p* < 0.05, NS indicates a non-significant difference. (TIF 244 kb) Additional file 4: Defensin C relative gene expression in the anterior midgut of *Rhodnius prolixus* fed on blood containing antibiotics and *Trypanosoma cruzi*. DefC gene expression in *R. prolixus* anterior midgut were analysed 7 days after feeding on inactivated blood containing antibiotics (ampicillin 300 μg/ml plus penicillin 300 μg/ml of blood) and *T. cruzi* Dm 28c clone at a final concentration of 1 × 10^7^ epimastigotes/mL. Treatments: black column-control insects fed only on blood; white column-insects fed on blood containing antibiotics; grid column-insects fed on blood containing *T. cruzi*; striped columns-insects fed on blood containing antibiotics and *T. cruzi*. Bars represent the mean ± SEM of 3 independent experiments with 3 pools of insects (*n* = 3). Means were compared using Student’s *T*-test; *** *p* < 0.001, ** *p* < 0.01, * *p* < 0.05. (TIF 403 kb) Additional file 5: Summary table of colony forming unit (CFU) and antibacterial activity against distinct Gram-negative and Gram-positive bacteria of *R. prolixus* midgut and haemolymph, 7 days after *T. cruzi* infection. (DOCX 12 kb) Additional file 6: Summary table of antimicrobial peptides (AMP) gene expression in *R. prolixus* midgut and fat body, 1 and 7 days after *T. cruzi* infection. (DOCX 13 kb)

## References

[1] Chagas C (1909). Nova tripanosomíase humana. Estudos sobre a morphologia e o ciclo evolutivo do *Schizotrypanum cruzi*, agente da nova entidade mórbida do homem. Mem Inst Oswaldo Cruz.

[2] Coura JR (2015). The main sceneries of Chagas disease transmission. The vectors, blood and oral transmissions-a comprehensive review. Mem Inst Oswaldo Cruz.

[3] WHO. Chagas disease (American trypanosomiasis) Fact sheet N° 340. http://www.who.int/mediacentre/factsheets/fs340/en/. Accessed 22 Oct 2015.

[4] Hashimoto K, Schofield CJ (2012). Elimination of *Rhodnius prolixus* in central America. Parasit Vectors.

[5] Wigglesworth V (1982). The principles of insect physiology.

[6] Azambuja P, Garcia ES, Ratcliffe NA (2005). Gut microbiota and parasite transmission by insect vectors. Trends Parasitol.

[7] Figueiredo MB, Castro DP, Nogueira NFS, Garcia ES, Azambuja P (2006). Cellular immune response in *Rhodnius prolixus*: role of ecdysone in hemocyte phagocytosis. J Insect Physiol.

[8] Garcia ES, Ratcliffe NA, Whitten MM, Gonzalez MS, Azambuja P (2007). Exploring the role of insect host factors in the dynamics of *Trypanosoma cruzi*-*Rhodnius prolixus* interactions. J Insect Physiol.

[9] Ursic-Bedoya R, Buchhop J, Joy JB, Durvasula R, Lowenberger C (2011). Prolixicin: a novel antimicrobial peptide isolated from *Rhodnius prolixus* with differential activity against bacteria and *Trypanosoma cruzi*. Insect Mol Biol.

[10] Castro D, Moraes C, Gonzalez M, Ratcliffe N, Azambuja P, Garcia E (2012). *Trypanosoma cruzi* immune response modulation decreases microbiota in *Rhodnius prolixus* gut and is crucial for parasite survival and development. PLoS One.

[11] Ribeiro JM, Genta FA, Sorgine MH, Logullo R, Mesquita RD, Paiva-Silva GO (2014). An insight into the transcriptome of the digestive tract of the bloodsucking bug, *Rhodnius prolixus*. PLoS Negl Trop Dis.

[12] Vieira CS, Waniek PJ, Mattos DP, Castro DP, Mello CB, Ratcliffe NA (2014). Humoral responses in *Rhodnius prolixus*: bacterial feeding induces differential patterns of antibacterial activity and enhances mRNA levels of antimicrobial peptides in the midgut. Parasit Vectors.

[13] Azambuja P, Garcia ES, Crampton JMBC, Louis C (1997). Care and maintenance of triatomine colonies. Molecular biology of insect disease vectors: a methods manual.

[14] Araújo CAC, Waniek PJ, Jansen AM (2009). An overview of Chagas disease and the role of triatomines on its distribution in Brazil. Vector Borne Zoonotic Dis.

[15] Coura JR, Viñas PA (2010). Chagas disease: a new worldwide challenge. Nature.

[16] Miles MA, Souza A, Povoa M, Shaw JJ, Lainson R, Toye PJ (1978). Isozymic heterogeneity of *Trypanosoma cruzi* in the first autochthonous patients with Chagas’ disease in Amazonian Brazil. Nature.

[17] Miles MA, Lanham SM, de Souza AA, Povoa M (1980). Further enzymic characters of *Trypanosoma cruzi* and their evaluation for strain identification. Trans R Soc Trop Med Hyg.

[18] Miles MA, Llewellyn MS, Lewis MD, Yeo M, Baleela R, Fitzpatrick S (2009). The molecular epidemiology and phylogeography of *Trypanosoma cruzi* and parallel research on *Leishmania*: looking back and to the future. Parasitology.

[19] Miles MA, Cedillos RA, Povoa MM, de Souza AA, Prata A, Macedo V (1981). Do radically dissimilar *Trypanosoma cruzi* strains (zymodemes) cause venezuelan and brazilian forms of Chagas’ disease?. Lancet.

[20] Zingales B, Andrade SG, Briones MR, Campbell DA, Chiari E, Fernandes O (2009). A new consensus for *Trypanosoma cruzi* intraspecific nomenclature: second revision meeting recommends TcI to TcVI. Mem Inst Oswaldo Cruz.

[21] Brener Z (1973). Biology of *Trypanosoma cruzi*. Annu Rev Microbiol.

[22] Garcia ES, Azambuja P (1991). Development and interactions of *Trypanosoma cruzi* within the insect vector. Parasitol Today.

[23] Azambuja P, Feder D, Garcia ES (2004). Isolation of *Serratia marcescens* in the midgut of *Rhodnius prolixus*: impact on the establishment of the parasite *Trypanosoma cruzi* in the vector. Exp Parasitol.

[24] Vallejo GA, Guhl F, Schaub GA (2009). Triatominae-*Trypanosoma cruzi*/*T. rangeli*: vector-parasite interactions. Acta Trop.

[25] Azambuja P, Feder D, Mello C, Gomes S, Garcia E (1999). Immunity in *Rhodnius prolixus*: trypanosomatid-vector interactions. Mem Inst Oswaldo Cruz.

[26] Steiner H, Hultmark D, Engstrom A, Bennich H, Boman HG (1981). Sequence and specificity of two antibacterial proteins involved in insect immunity. Nature.

[27] Ferrandon D, Imler JL, Hetru C, Hoffmann JA (2007). The *Drosophila* systemic immune response: sensing and signalling during bacterial and fungal infections. Nat Rev Immunol.

[28] Lamberty M, Zachary D, Lanot R, Bordereau C, Robert A, Hoffmann JA (2001). Insect immunity. Constitutive expression of a cysteine-rich antifungal and a linear antibacterial peptide in a termite insect. J Biol Chem.

[29] Bulet P, Stocklin R (2005). Insect antimicrobial peptides: structures, properties and gene regulation. Protein Pept Lett.

[30] Charroux B, Royet J (2010). *Drosophila* immune response: from systemic antimicrobial peptide production in fat body cells to local defense in the intestinal tract. Fly.

[31] Garcia ES, Genta FA, de Azambuja P, Schaub GA (2010). Interactions between intestinal compounds of triatomines and *Trypanosoma cruzi*. Trends Parasitol.

[32] Castro DP, Seabra SH, Garcia ES, de Souza W, Azambuja P (2007). *Trypanosoma cruzi*: ultrastructural studies of adhesion, lysis and biofilm formation by *Serratia marcescens*. Exp Parasitol.

[33] Laboratório SBdCdAe. COBEA. http://www.cobea.org.br. Accessed 22 Oct 2015.

[34] Contreras VT, Araújo-Jorge TC, Bonaldo MC, Thomaz N, Barbosa HS, Meirelles Mde N (1988). Biological aspects of the Dm 28c clone of *Trypanosoma cruzi* after metacyclogenesis in chemically defined media. Mem Inst Oswaldo Cruz.

[35] Silva LHP, Nussenzweig V (1953). Sôbre uma cepa de *Trypanosoma cruzi* altamente virulenta para o camundongo branco. Folia Clin Biol.

[36] Vieira CS, Mattos DP, Waniek PJ, Santangelo JM, Figueiredo MB, Gumiel M (2015). *Rhodnius prolixus* interaction with *Trypanosoma rangeli*: modulation of the immune system and microbiota population. Parasit Vectors.

[37] Lopez L, Morales G, Ursic R, Wolff M, Lowenberger C (2003). Isolation and characterization of a novel insect defensin from *Rhodnius prolixus*, a vector of Chagas disease. Insect Biochem Mol Biol.

[38] Paim RM, Pereira MH, Di Ponzio R, Rodrigues JO, Guarneri AA, Gontijo NF (2012). Validation of reference genes for expression analysis in the salivary gland and the intestine of *Rhodnius prolixus* (Hemiptera, Reduviidae) under different experimental conditions by quantitative real-time PCR. BMC Res Notes.

[39] Livak KJ, Schmittgen TD (2001). Analysis of relative gene expression data using real-time quantitative PCR and the 2(−delta delta C(T)) method. Methods.

[40] Da Mota FF, Marinho LP, Moreira CJ, Lima MM, Mello CB, Garcia ES (2012). Cultivation-independent methods reveal differences among bacterial gut microbiota in triatomine vectors of Chagas disease. PLoS Negl Trop Dis.

[41] Gumiel M, da Mota FF, Rizzo Vde S, Sarquis O, de Castro DP, Lima MM (2015). Characterization of the microbiota in the guts of *Triatoma brasiliensis* and *Triatoma pseudomaculata* infected by *Trypanosoma cruzi* in natural conditions using culture independent methods. Parasit Vectors.

[42] Eichler S, Schaub GA (2002). Development of symbionts in triatomine bugs and the effects of infections with trypanosomatids. Exp Parasitol.

[43] Mesquita RD, Vionette-Amaral RJ, Lowenberger C, Rivera-Pomar R, Monteiro FA, Minx P et al. Genome of *Rhodnius prolixus*, an insect vector of Chagas disease, reveals unique adaptations to hematophagy and parasite infection. Proc Natl Acad Sci USA. 2015;12:14936–41. 10.1073/pnas.1506226112 PMC4672799 26627243

[44] Weiss B, Aksoy S (2011). Microbiome influences on insect host vector competence. Trends Parasitol.

[45] Gendrin M, Christophides G, Manguin S (2013). The *Anopheles* mosquito microbiota and their impact on pathogen transmission. *Anopheles* mosquitoes—New insights into malaria vectors.

[46] Dong Y, Manfredini F, Dimopoulos G (2009). Implication of the mosquito midgut microbiota in the defense against malaria parasites. PLoS Pathog.

[47] Meister S, Agianian B, Turlure F, Relogio A, Morlais I, Kafatos FC (2009). *Anopheles gambiae* PGRPLC-mediated defense against bacteria modulates infections with malaria parasites. PLoS Pathog.

[48] Cirimotich CM, Dong Y, Clayton AM, Sandiford SL, Souza-Neto JA, Mulenga M (2011). Natural microbe-mediated refractoriness to *Plasmodium* infection in *Anopheles gambiae*. Science.

[49] Boulanger N, Lowenberger C, Volf P, Ursic R, Sigutova L, Sabatier L (2004). Characterization of a defensin from the sand fly *Phlebotomus duboscqi* induced by challenge with bacteria or the protozoan parasite *Leishmania major*. Infect Immun.

[50] Telleria EL, Sant’Anna MR, Alkurbi MO, Pitaluga AN, Dillon RJ, Traub-Csekö YM (2013). Bacterial feeding, *Leishmania* infection and distinct infection routes induce differential defensin expression in *Lutzomyia longipalpis*. Parasit Vectors.

[51] Soares TS, Buarque DS, Queiroz BR, Gomes CM, Braz GR, Araujo RN (2015). A kazal-type inhibitor is modulated by *Trypanosoma cruzi* to control microbiota inside the anterior midgut of *Rhodnius prolixus*. Biochimie.

[52] Boulanger N, Ehret-Sabatier L, Brun R, Zachary D, Bulet P, Imler JL (2001). Immune response of *Drosophila melanogaster* to infection with the flagellate parasite *Crithidia spp*. Insect Biochem Mol Biol.

[53] Boulanger N, Brun R, Ehret-Sabatier L, Kunz C, Bulet P (2002). Immunopeptides in the defense reactions of *Glossina morsitans* to bacterial and *Trypanosoma brucei brucei* infections. Insect Biochem Mol Biol.

[54] Hao Z, Kasumba I, Lehane MJ, Gibson WC, Kwon J, Aksoy S (2001). Tsetse immune responses and trypanosome transmission: implications for the development of tsetse-based strategies to reduce trypanosomiasis. Proc Natl Acad Sci USA.

[55] Hao Z, Kasumba I, Aksoy S (2003). Proventriculus (cardia) plays a crucial role in immunity in tsetse fly (Diptera: Glossinidiae). Insect Biochem Mol Biol.

[56] Cortez MR, Provencano A, Silva CE, Mello CB, Zimmermann LT, Schaub GA (2012). *Trypanosoma cruzi*: effects of azadirachtin and ecdysone on the dynamic development in *Rhodnius prolixus* larvae. Exp Parasitol.

[57] Tzou P, Ohresser S, Ferrandon D, Capovilla M, Reichhart JM, Lemaitre B (2000). Tissue-specific inducible expression of antimicrobial peptide genes in *Drosophila* surface epithelia. Immunity.

[58] Dimopoulos G, Seeley D, Wolf A, Kafatos FC (1998). Malaria infection of the mosquito *Anopheles gambiae* activates immune-responsive genes during critical transition stages of the parasite life cycle. EMBO J.

[59] Araújo CAC, Waniek PJ, Stock P, Mayer C, Jansen AM, Schaub GA (2006). Sequence characterization and expression patterns of defensin and lysozyme encoding genes from the gut of the reduviid bug *Triatoma brasiliensis*. Insect Biochem Mol Biol.

[60] Waniek PJ, Castro HC, Sathler PC, Miceli L, Jansen AM, Araújo CAC (2009). Two novel defensin-encoding genes of the Chagas disease vector *Triatoma brasiliensis* (Reduviidae, Triatominae): gene expression and peptide-structure modeling. J Insect Physiol.

[61] Araújo CAC, Lima AC, Jansen AM, Galvão C, Jurberg J, Costa J (2015). Genes encoding defensins of important Chagas disease vectors used for phylogenetic studies. Parasitol Res.

[62] Hu Y, Aksoy S (2005). An antimicrobial peptide with trypanocidal activity characterized from *Glossina morsitans morsitans*. Insect Biochem Mol Biol.

[63] Hu C, Aksoy S (2006). Innate immune responses regulate trypanosome parasite infection of the tsetse fly *Glossina morsitans morsitans*. Mol Microbiol.

[64] De Souza W (1995). Structural organization of the cell surface of pathogenic protozoa. Micron.

[65] Araújo CAC, Mello CB, Jansen AM (2002). *Trypanosoma cruzi* I and *Trypanosoma cruzi* II: recognition of sugar structures by *Arachis hypogaea* (peanut agglutinin) lectin. J Parasitol.

[66] Burgos JM, Risso MG, Breniere SF, Barnabe C, Campetella O, Leguizamon MS (2013). Differential distribution of genes encoding the virulence factor trans-sialidase along *Trypanosoma cruzi* discrete typing units. PLoS One.

[67] Gonzalez MS, Souza MS, Garcia ES, Nogueira NF, Mello CB, Canepa GE (2013). *Trypanosoma cruzi* TcSMUG L-surface mucins promote development and infectivity in the triatomine vector *Rhodnius prolixus*. PLoS Negl Trop Dis.

[68] Abel LC, Ferreira LR, Cunha Navarro I, Baron MA, Kalil J, Gazzinelli RT (2014). Induction of IL-12 production in human peripheral monocytes by *Trypanosoma cruzi* is mediated by glycosylphosphatidylinositol-anchored mucin-like glycoproteins and potentiated by IFN-γ and CD40-CD40L interactions. Mediators Inflamm.

[69] Freire-de-Lima L, Fonseca LM, Oeltmann T, Mendonça-Previato L, Previato JO (2015). The trans-sialidase, the major *Trypanosoma cruzi* virulence factor: three decades of studies. Glycobiology.

[70] Schocker NS, Portillo S, Brito CR, Marques AF, Almeida IC, Michael K (2016). Synthesis of galα(1,3)galβ(1,4)GlcNAcα-, galβ(1,4)GlcNAcα- and GlcNAcα-containing neoglycoproteins and their immunological evaluation in the context of Chagas disease. Glycobiology.

[71] Araújo CAC, Waniek PJ, Xavier SC, Jansen AM (2011). Genotype variation of *Trypanosoma cruzi* isolates from different Brazilian biomes. Exp Parasitol.

[72] Araújo CAC, Waniek PJ, Jansen AM (2014). TcI/TcII co-infection can enhance *Trypanosoma cruzi* growth in *Rhodnius prolixus*. Parasit Vectors.

[73] Hurwitz I, Fieck A, Read A, Hillesland H, Klein N, Kang A (2011). Paratransgenic control of vector borne diseases. Int J Biol Sci.

[74] Taracena ML, Oliveira PL, Almendares O, Umana C, Lowenberger C, Dotson EM (2015). Genetically modifying the insect gut microbiota to control Chagas disease vectors through systemic RNAi. PLoS Negl Trop Dis.

