# Supplementary material for: Impact of Trypanosoma cruzi on antimicrobial peptide gene expression and activity in the fat body and midgut of Rhodnius prolixus
Source: Parasit Vectors. 2016 Mar 1;9:119. doi: 10.1186/s13071-016-1398-4 (PMC4774030; doi:10.1186/s13071-016-1398-4)
Supplement: Additional file 1: — Oligonucleotide primers used for qPCR analysis. (DOC 46 kb) [file 13071_2016_1398_MOESM1_ESM.doc]

**Table**

**Additional file 1:** Oligonucleotide primers used for qPCR analysis

| Gene/name | Sequence 5’-3’ | Amplicon length | Reference |
| --- | --- | --- | --- |
| GAPDH-F | GATGGCGCCCAGTACATAGT |  |  |
| GAPDH-R | AGCTGACGGGGCTGTTATTA | 111 bp | Paim et al., 2012 |
| TUB-F | TTTCCTCGATCACTGCTTCC |  |  |
| TUB-R | CGGAAATAACTGGGGCATAA | 129 bp | Paim et al., 2012 |
| RPDEFA-F | GAATACTCCACTCAACCGCAAC |  |  |
| RPDEFA-R | agggcatcatctagttgttgatgagtg | 131 bp | Present study |
| RPDEFB-F | GGATATTCCACTCAACCGCAAC |  |  |
| RPDEFB-R | agagcatcgtctaattcttgttgagtg | 131 bp | Present study |
| RPDEFC-F | CAGTACAGTCCTAATACCTAGCC |  |  |
| RPDEFC-R | tgggcatcatctaattgatgttgagaa | 136 bp | Present study |
| F1 (prolixicin) | ACAATTTTGGTGGTGGTTGTC |  |  |
| qR (prolixicin) | GCTTGAGCTCTGGTCCTTCC | 194 bp | Ursic-Bedoya et al., 2011 |
| SmarF | GGTGAGCTTAATACGTTCATCAATTG |  |  |
| SmarR | GCAGTTCCCAGGTTGAGCC | 179 bp | Saikaly et al., 2007 |
| RrhoF | CACTGGTTGCATGGCCTGGTG |  |  |
| RrhoR | TGAGCTGTGGGATTTCACAGAC | 418 bp | Present study |
