# Supplementary figures and images for: Impact of Trypanosoma cruzi on antimicrobial peptide gene expression and activity in the fat body and midgut of Rhodnius prolixus
Source: Parasit Vectors. 2016 Mar 1;9:119. doi: 10.1186/s13071-016-1398-4 (PMC4774030; doi:10.1186/s13071-016-1398-4)

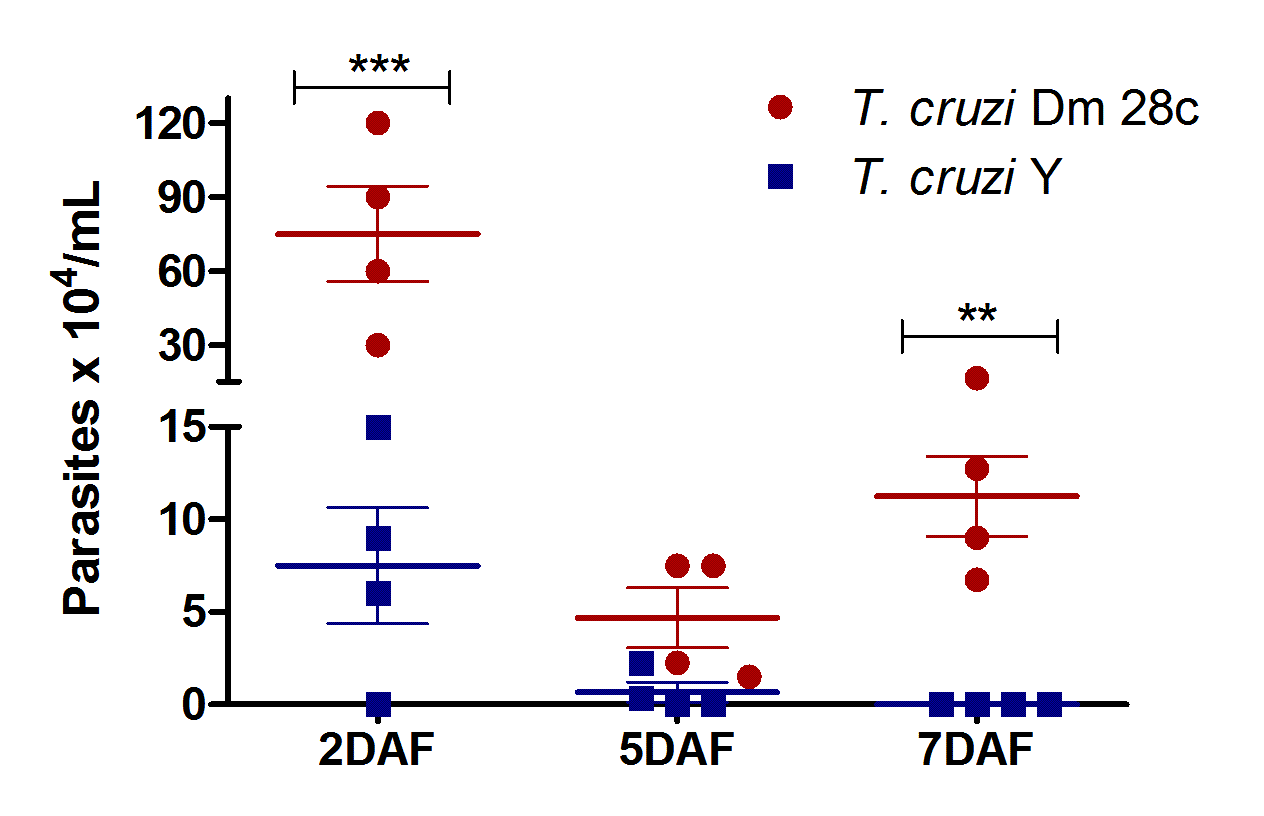

Supplement: Additional file 2: — Parasite population in Rhodnius prolixus digestive tract. The numbers of T. cruzi Dm 28c and Y strain parasites were estimated in the whole digestive tracts of R. prolixus 5th instar nymphs at different days after feeding (DAF). Each point represents the number of parasites in an individual insect, and bars indicate the median. Means were compared using Student’s T-test or Mann-Whitney test; *** p < 0.001, * p < 0.05. (BMP 64 kb) [file 13071_2016_1398_MOESM2_ESM.bmp]

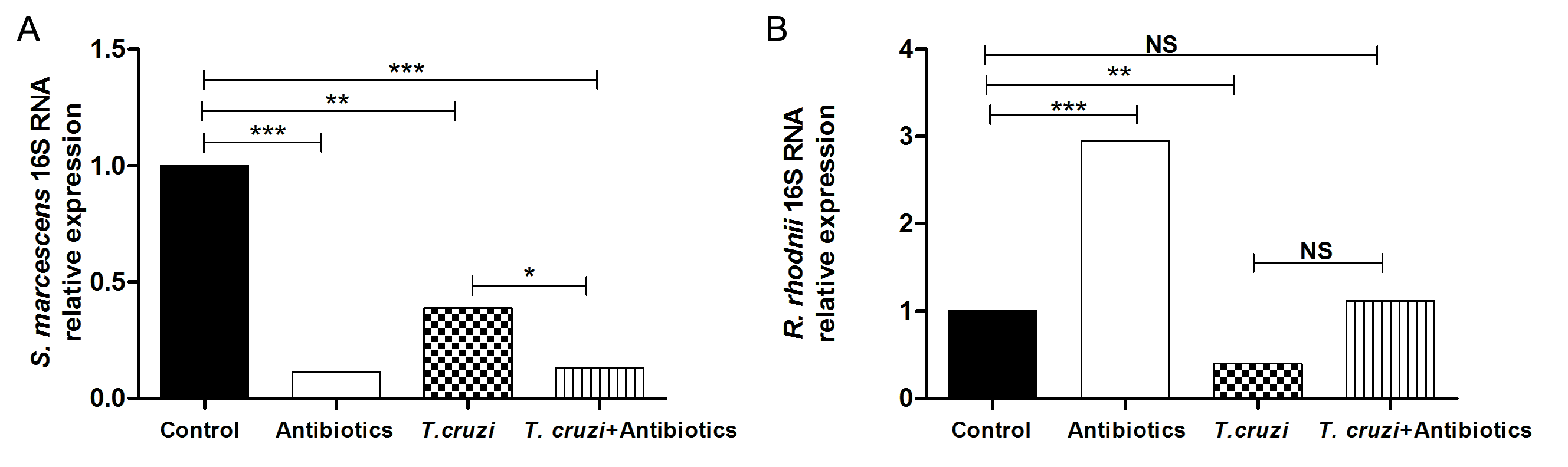

Supplement: Additional file 3: — Determination of bacterial load in the anterior midgut of Rhodnius prolixus after antibiotic treatment and Trypanosoma cruzi Dm 28c infection by RT-qPCR. R. prolixus anterior midgut were analysed 7 days after feeding on inactivated blood containing antibiotics (ampicillin 300 μg/ml plus penicillin 300 μg/ml of blood) and T. cruzi Dm 28c clone at a final concentration of 1 × 10^7^ epimastigotes/mL. Relative expression of 16S-rRNAS of A−Serratia marcescens B−Rhodococcus rhodnii. Treatments: black column—control insects fed only on blood; white column-insects fed on blood containing antibiotics; grid column-insects fed on blood containing T. cruzi; striped columns-insects fed on blood containing antibiotics and T. cruzi. Two biological samples in triplicate were used for each group. All data were normalized to the R. prolixus α-tubulin, representing the mean of identical triplicates ± standard error. Bars represent the mean ± SEM of 3 independent experiments with 3 pools of insects (n = 3). Means were compared using Student’s T-test; *** p < 0.001, ** p < 0.01, * p < 0.05, NS indicates a non-significant difference. (TIF 244 kb) [file 13071_2016_1398_MOESM3_ESM.tif]

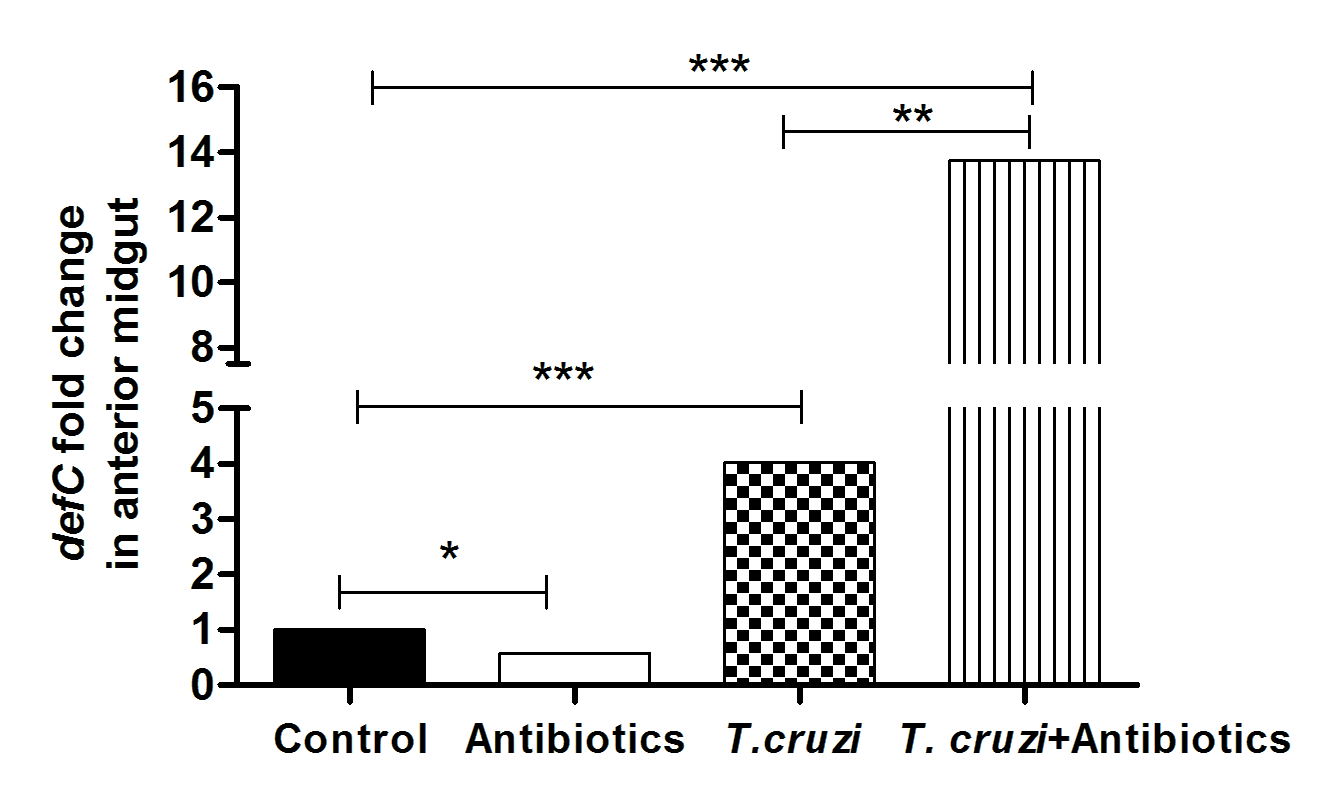

Supplement: Additional file 4: — Defensin C relative gene expression in the anterior midgut of Rhodnius prolixus fed on blood containing antibiotics and Trypanosoma cruzi. DefC gene expression in R. prolixus anterior midgut were analysed 7 days after feeding on inactivated blood containing antibiotics (ampicillin 300 μg/ml plus penicillin 300 μg/ml of blood) and T. cruzi Dm 28c clone at a final concentration of 1 × 10^7^ epimastigotes/mL. Treatments: black column-control insects fed only on blood; white column-insects fed on blood containing antibiotics; grid column-insects fed on blood containing T. cruzi; striped columns-insects fed on blood containing antibiotics and T. cruzi. Bars represent the mean ± SEM of 3 independent experiments with 3 pools of insects (n = 3). Means were compared using Student’s T-test; *** p < 0.001, ** p < 0.01, * p < 0.05. (TIF 403 kb) [file 13071_2016_1398_MOESM4_ESM.tif]
