# Supplementary material for: Impact of Trypanosoma cruzi on antimicrobial peptide gene expression and activity in the fat body and midgut of Rhodnius prolixus
Source: Parasit Vectors. 2016 Mar 1;9:119. doi: 10.1186/s13071-016-1398-4 (PMC4774030; doi:10.1186/s13071-016-1398-4)
Supplement: Additional file 5: — Summary table of colony forming unit (CFU) and antibacterial activity against distinct Gram-negative and Gram-positive bacteria of R. prolixus midgut and haemolymph, 7 days after T. cruzi infection. (DOCX 12 kb) [file 13071_2016_1398_MOESM5_ESM.docx]

**Additional file 5:** Summary table of colony forming unit (CFU) and antibacterial activity against distinct Gram-negative and Gram-positive bacteria of *R. prolixus* midgut and haemolymph, 7 days after *T. cruzi* infection.

|  | |  | |  | | **Antibacterial activity** | | | |  | |
| --- | --- | --- | --- | --- | --- | --- | --- | --- | --- | --- | --- |
| ***T. cruzi*** | | **CFU** | | **Haemolymph** | | **Anterior**  **midgut** | | | **Posterior midgut** | | |
|  | **AM** | | **PM** | **E.c.** | **S.a.** | **E.c.** | **S.a.** | **S.m.** | | **E.c.** | **S.a.** |
| **Dm28c** | **↓** | | **↑** | **↑** | **↑** | **−** | **−** | **↑** | | **↑** | **−** |
| **Y** | **↓** | | **−** | **↑** | **↑** | **−** | **↓** | **−** | | **−** | **−** |

AM = anterior midgut; PM= posterior midgut; E.c. = *Escherichia coli*; S. a. = *Staphylococcus aureus*; S.m. = *Serratia marcescens*; ↓ = significant decrease; ↑ = significant increase; − no significant change.
