# Supplementary material for: Impact of Trypanosoma cruzi on antimicrobial peptide gene expression and activity in the fat body and midgut of Rhodnius prolixus
Source: Parasit Vectors. 2016 Mar 1;9:119. doi: 10.1186/s13071-016-1398-4 (PMC4774030; doi:10.1186/s13071-016-1398-4)
Supplement: Additional file 6: — Summary table of antimicrobial peptides (AMP) gene expression in R. prolixus midgut and fat body, 1 and 7 days after T. cruzi infection. (DOCX 13 kb) [file 13071_2016_1398_MOESM6_ESM.docx]

**Additional file 6:** Summary table of antimicrobial peptides (AMP) gene expression in *R. prolixus* midgut and fat body, 1 and 7 days after *T. cruzi* infection.

| ***T. cruzi*** |  | ***DefA*** | | | ***DefB*** | | | ***DefC*** | | | ***Prol*** | | |
| --- | --- | --- | --- | --- | --- | --- | --- | --- | --- | --- | --- | --- | --- |
|  | **DAF** | **AM** | **PM** | **FB** | **AM** | **PM** | **FB** | **AM** | **PM** | **FB** | **AM** | **PM** | **FB** |
| **Dm 28c** | **1** | **−** | **ND** | **↓** | **↑** | **ND** | **−** | **−** | **−** | **↑** | **↓** | **↑** | **−** |
|  | **7** | **−** | **ND** | **↑** | **ND** | **ND** | **−** | **↑** | **↑** | **↑** | **↑** | **−** | **↑** |
| **Y** | **1** | **−** | **ND** | **↓** | **↓** | **ND** | **↓** | **−** | **−** | **↑** | **−** | **−** | **↓** |
|  | **7** | **↓** | **ND** | **−** | **ND** | **ND** | **−** | **−** | **↓** | **−** | **−** | **−** | **↑** |

AM = anterior midgut; PM= posterior midgut; FB = fat body; ↓ = significant decrease; ↑ = significant increase; − no significant change; ND = not detected; Def = defensin encoding genes A-C; Prol = prolixicin encoding gene; DAF = days after feeding.
